# Enhanced Antitumor Effects of Epidermal Growth Factor Receptor Targetable Cetuximab-Conjugated Polymeric Micelles for Photodynamic Therapy

**DOI:** 10.3390/nano8020121

**Published:** 2018-02-22

**Authors:** Ming-Hsiang Chang, Chin-Ling Pai, Ying-Chen Chen, Hsiu-Ping Yu, Chia-Yen Hsu, Ping-Shan Lai

**Affiliations:** 1 Department of Chemistry, National Chung Hsing University, Taichung 402, Taiwan; s19850309@gmail.com (M.-H.C.); ritachen0623@gmail.com (Y.-C.C.); hsiupingyu0126@gmail.com (H.-P.Y.); clhsqoo@gmail.com (C.-Y.H.); 2 Ph.D. Program in Tissue Engineering and Regenerative Medicine, National Chung Hsing University, Taichung 402, Taiwan; pailittleelf@gmail.com

**Keywords:** photodynamic therapy, polymeric micelles, C225, EGF receptor, in vivo

## Abstract

Nanocarrier-based delivery systems are promising strategies for enhanced therapeutic efficacy and safety of toxic drugs. Photodynamic therapy (PDT)—a light-triggered chemical reaction that generates localized tissue damage for disease treatments—usually has side effects, and thus patients receiving photosensitizers should be kept away from direct light to avoid skin phototoxicity. In this study, a clinically therapeutic antibody cetuximab (C225) was conjugated to the surface of methoxy poly(ethylene glycol)-*b*-poly(lactide) (mPEG-*b*-PLA) micelles via thiol-maleimide coupling to allow tumor-targetable chlorin e6 (Ce6) delivery. Our results demonstrate that more C225-conjugated Ce6-loaded polymeric micelles (C225-Ce6/PM) were selectively taken up than Ce6/PM or IgG conjugated Ce6/PM by epidermal growth factor receptor (EGFR)-overexpressing A431 cells observed by confocal laser scanning microscopy (CLSM), thereby decreasing the IC_50_ value of Ce6-mediated PDT from 0.42 to 0.173 μM. No significant differences were observed in cellular uptake study or IC_50_ value between C225-Ce6/PM and Ce6/PM groups in lower EGFR expression HT-29 cells. For antitumor study, the tumor volumes in the C225-Ce6/PM-PDT group (percentage of tumor growth inhibition, TGI% = 84.8) were significantly smaller than those in the Ce6-PDT (TGI% = 38.4) and Ce6/PM-PDT groups (TGI% = 53.3) (*p* < 0.05) at day 21 through reduced cell proliferation in A431 xenografted mice. These results indicated that active EGFR targeting of photosensitizer-loaded micelles provides a possible way to resolve the dose-limiting toxicity of conventional photosensitizers and represents a potential delivery system for PDT in a clinical setting.

## 1. Introduction

Photodynamic therapy (PDT), based on the photosensitizer-light-oxygen triad, is a type of minimally invasive phototherapy involving cascaded reactions that result in local cytotoxic damage by reactive oxygen species (ROS) following the excitation of the photosensitizer with a specific light source. PDT is currently utilized for the treatment of cancer and various nonmalignant conditions [1]. The major side effect of clinical PDT is skin phototoxicity in healthy tissues, and patients receiving photosensitizers must avoid direct sunlight for several weeks [2]. Many photosensitizers are highly hydrophobic, and thus the formulation is very important for drug development. It is known that the aggregation of hydrophobic photosensitizers in aqueous solution results in less photoactivation and poor ROS generation in solution, thereby subsequently reducing the efficacy of PDT against tumor cells [3]. Many hydrophobic photosensitizers used in current clinical studies are formulated using surfactants such as Cremophor and Tween-80 as delivery agents; however, hypersensitivity and toxicity issues could be a concern for multiple-dose treatments [4,5]. Thus, a suitable nanocarrier for the delivery of hydrophobic photosensitizers that can selectively enhance the concentration of the photosensitizer in tumor areas is necessary to improve the PDT effects and reduce skin photosensitivity based on the optimization of the “drug-light-oxygen triad”.

Recently, nanotechnology-based drug delivery systems such as polymer-drug conjugates [6,7], liposomes [8,9,10], artificial oil bodies [11], nanoparticles [12,13], polymeric micelles [14], polymeric vesicles, and polymersomes [15] have been demonstrated to be excellent platforms for hydrophobic drug delivery. For the delivery of hydrophobic photosensitizers, polymeric carriers have emerged as attractive agents that avoid the aforementioned problems associated with photosensitizers [16,17,18,19,20]. In addition, nanocarriers may increase the accumulation of cytotoxic agents in tumor tissues based on enhanced permeability and retention (EPR) effects [21]. In our previous studies, several nanocarriers were demonstrated to be efficient in PDT. For example, porphyrin/chlorin-core star-shaped block copolymers can self-assemble to form micelle structures for dual PDT/chemotherapy [22]. Porphyrin-polylactide star polymers can self-assemble to form unique photosensitizer-embedded vesicles that can encapsulate gases and be used as ultrasonic imaging/PDT cancer theranostic agents [23]. Compared with EPR effect-based passive targeting nanocarriers, active targeting photosensitizers have been suggested to enhance the selective accumulation of drugs in the tumor area, resulting in enhanced photoactivation and more effective PDT. Based on the tumor via targeting ligand/drug or ligand/carrier combinations, the antitumor efficacy of nanomedicines was augmented [24,25,26].

Epidermal growth factor receptor (EGFR) is a 170-kDa transmembrane glycoprotein with an intracellular tyrosine kinase domain that is significantly overexpressed on the surface of cancer cells such as glioblastomas and breast, colon, bladder, ovarian, prostate, renal, gastric, and lung carcinomas, and has been utilized as an important target in actively targetable cancer therapy [27]. For example, cetuximab (C225)—an effective chimeric monoclonal antibody—has been used in clinical cancer treatments that target human EGFR and inhibits EGFR-dependent primary tumor growth and metastasis [28]. Recently, EGFR has been used as a target or mediator of ligand-nanoparticle or ligand-drug conjugates for drug delivery [29,30]. In this study, we attempted to increase the tumor specificity and therapeutic efficacy of photosensitizer-loaded micelle-mediated PDT by C225 conjugation. The second-generation photosensitizer chlorin e6 (Ce6) with improved efficacy, decreased side effects, and longer wavelength for photoactivation compared with the first-generation one was used for PDT [1]. The C225-conjugated micelles may interact with EGFR, which would not only enhance the internalization and accumulation of the photosensitizer in cancer cells, but also directly inhibit tumor growth. The antitumor efficacy of Ce6-loaded methoxy poly(ethylene glycol)-*b*-poly(lactide) (mPEG-*b*-PLA) micelles conjugated to C225 was evaluated in EGFR-overexpressing A431 human epidermoid cancer in vitro and in vivo.

## 2. Results

### 2.1. Characterization of Chlorin e6-Loaded Micelles with or without C225 Conjugation

**Figure 1:**
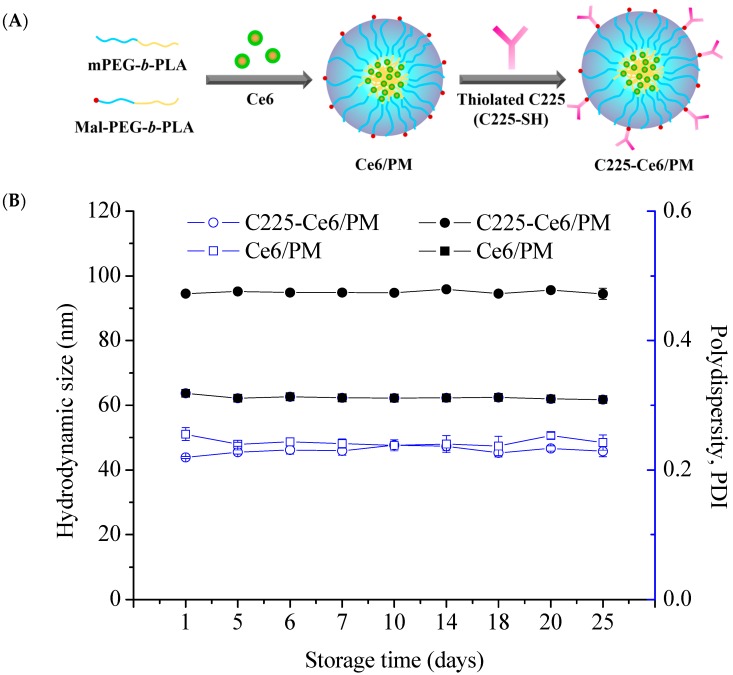
(**A**) Schematic representation of chlorin e6 (Ce6)-loaded micelles and cetuximab (C225)-conjugated Ce6-loaded polymeric micelles (C225-Ce6/PM); (**B**) Change in the particle size and PDI of Ce6/PM and C225-Ce6/PM over time at 4 °C measured by dynamic light scattering DLS.

The encapsulation of photosensitizer chlorin e6 (Ce6) was performed using mPEG-*b*-PLA micelles or mPEG-*b*-PLA/ maleimide (Mal)-PEG-*b*-PLA mixed micelles via the solvent evaporation method (Figure 1A). At a 1:10 feed ratio of Ce6 to polymer, 97% of the Ce6 was loaded into micelles with average diameters of approximately 65.0 nm (polydispersity index, PDI = 0.24). For C225-conjugated Ce6-loaded polymeric micelles (C225-Ce6/PM) preparation, Ce6 was first loaded into mixed micelles, and the resulting micelles were then reacted with thiolated C225 at 4 °C. The purification procedure of C225-Ce6/PM using a Sepharose CL-4B column is presented in Figure S1, and the purity was confirmed using sodium dodecyl sulfate polyacrylamide gel electrophoresis (SDS-PAGE) analysis with Coomassie brilliant blue staining and UV light exposure (Figure S2). These results indicate that the thiolated C225 was successfully conjugated to the surface of the micelles [31] and that the free thiolated C225 could be removed by CL-4B gel filtration chromatography [32]. It was noted that a clear band could be observed during the separation process, indicating the existence of Ce6 in the micelles. The hydrodynamic size of C225-Ce6/PM was approximately 91.6 nm with a PDI of 0.25, as determined by dynamic light scattering (DLS, ZS 90, Malvern Instruments Ltd., Worcestershire, UK). Obviously, C225 conjugation to the micelle surface significantly increased the hydrodynamic size of the Ce6-loaded micelles. IgG-conjugated Ce6-loaded micelles were prepared using a protocol similar to that for C225-Ce6/PM, and the resulting IgG-Ce6/PM had a particle size of 94.5 nm with a PDI of 0.24. The conjugation numbers of C225 or IgG on the micellar surface were 18 and 17, determined as in a previous report [33], and the encapsulation efficiencies of Ce6 in C225-Ce6/PM and IgG-Ce6/PM were 88% and 86%, respectively. The C225-conjugated mixed micelle stability was evaluated in 10% (*v*/*v*) serum, and negligible Ce6 release was observed at pH 7.4. As shown in Figure 1B, the size and PDI of the Ce6-loaded micelles—including C225-Ce6/PM—did not change significantly over a period of 25 days at 4 °C without precipitation, indicating highly stability of C225-Ce6/PM.

### 2.2. Cellular Uptake of Ce6-Loaded Micelles with or without C225 Conjugation

**Figure 2:**
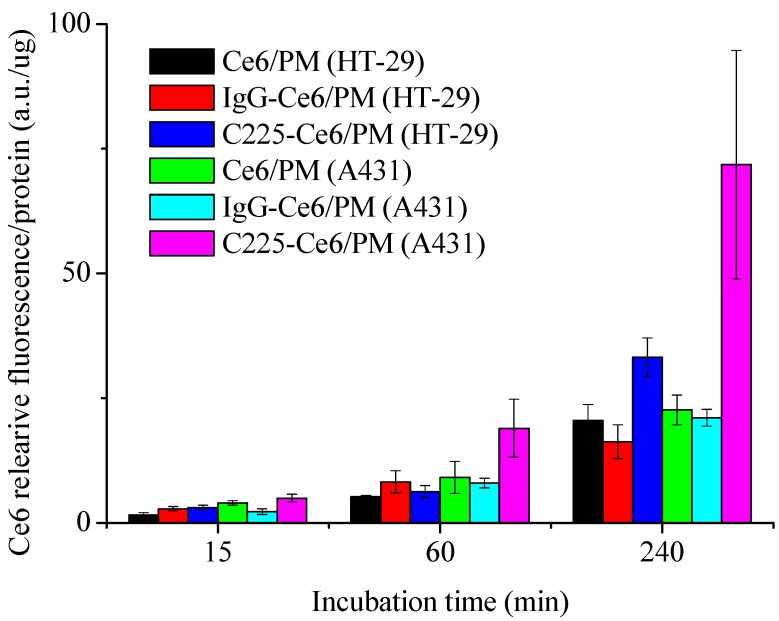
The quantitative fluorescence levels of Ce6/PM, IgG-Ce6/PM, and C225-Ce6/PM taken up by A431 or HT-29 cells at a constant photosensitizer concentration for different incubation times.

To assess the selectivity and cellular uptake of C225-Ce6/PM by EGFR-overexpressing tumor cells, the intracellular fluorescence signals of the photosensitizer were assessed by spectrofluorimetric methods and CLSM (Leica-SP5, Leica Microsystems Heidelberg GmbH, Heidelberg, Germany). Figure 2 shows the quantitative fluorescence levels of Ce6/PM, IgG-Ce6/PM, and C225-Ce6/PM taken up by A431 or HT-29 cells at a constant photosensitizer concentration for different incubation times. After a 15-min incubation, similar amounts of Ce6/PM, IgG-Ce6/PM, and C225-Ce6/PM were accumulated in high-EGFR-expressing A431 cells. After 1 or 4 h of incubation, higher Ce6 fluorescence intensity of C225-Ce6/PM was significantly detected in A431 cells, whereas non-targeted micelles including Ce6/PM and IgG-Ce6/PM revealed similar low fluorescence level in A431 cells. Obviously, the fluorescence intensity of Ce6 using the C225-conjugated delivery system in A431 cells was 2.16-fold higher than that in low-EGFR-expressing HT-29 cells at 4 h post-treatments, indicating the possibility of EGFR-mediated internalization in C225-Ce6/PM. Figure S3 shows the uptake of Ce6/PM and C225-Ce6/PM by A431 or HT-29 cells at a constant photosensitizer concentration for 1 h with or without C225 pretreatment. Cytosolic fluorescent dots were readily observed in A431 cells incubated with C225-Ce6/PM (Figure S3C), whereas cells incubated with Ce6/PM exhibited lower fluorescence intensity (Figure S3A). Cells pretreated with free C225 had significantly lower fluorescence intensities for Ce6, indicating that the excess free C225 in the medium prevented the uptake of C225-Ce6/PM into A431 cells as the result of competitive binding to EGFR on the cell surface (Figure S3D). For low-EGFR-expressing HT-29 cells, lower intracellular fluorescence intensities for Ce6 were observed in Ce6/PM and C225-Ce6/PM groups with or without free C225 pretreatment (Figure S3E–H). The polymeric micelles not only provided a relatively large drug reservoir for the hydrophobic photosensitizer, but also provided specificity to EGFR-overexpressing cancer cells. Thus, the results in Figure 2 and Figure S3 suggested that C225-Ce6/PM can deliver more Ce6 into EGFR-expressing cells, and free C225 in the medium can prevent the uptake of C225-Ce6/PM into cells as the result of competitive binding to EGFR on the cell surface.

**Figure 3:**
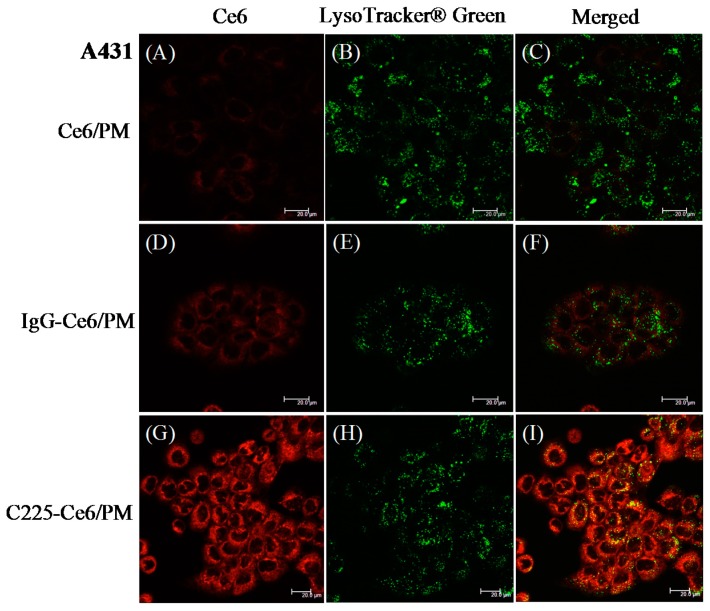
Confocal images of high-epidermal growth factor receptor (EGFR)-expressing A431 cells treated with Ce6/PM, IgG-Ce6/PM or C225-Ce6/PM for 240 min. Cells were treated with LysoTracker Green to visualize acidic endosomes/lysosomes. The Ce6, LysoTracker Green and merged fluorescence images of (**A**–**C**) Ce6/PM; (**D**–**F**) IgG-Ce6/PM and (**G**–**I**) C225-Ce6/PM treated A431 cells, respectively. Scale bar: 20 µm.

**Figure 4:**
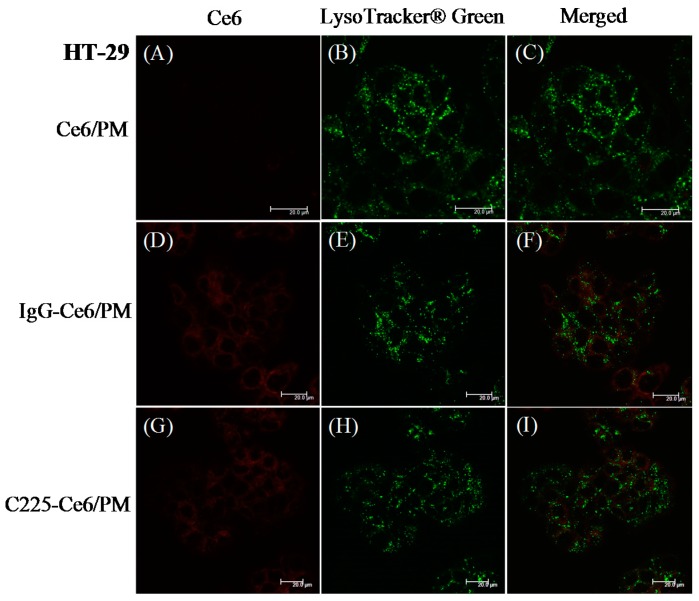
Confocal images of low-EGFR-expressing HT29 cells treated with Ce6/PM, IgG-Ce6/PM or C225-Ce6/PM for 240 min. Cells were treated with LysoTracker Green to visualize acidic endosomes/lysosomes. The Ce6, LysoTracker Green and merged fluorescence images of (**A**–**C**) Ce6/PM; (**D**–**F**) IgG-Ce6/PM and (**G**–**I**) C225-Ce6/PM treated HT29 cells, respectively. Scale bar: 20 µm.

It is demonstrated that C225 can induce EGFR-mediated endocytosis via dynamin-dependent and dynamin-independent pathways in different cell types [29]. To determine whether the Ce6 delivered by C225-conjugated micelles was internalized via the endocytic pathway, A431 cells Figure 3 or HT-29 cells Figure 4 treated with Ce6/PM, IgG/PM, or C225-Ce6/PM were stained using LysoTracker Green DND-26 (LysoTracker Green, Molecular Probe, Leiden, The Netherlands) and then observed by CLSM. For Ce6/PM or IgG-Ce6/PM, only a low level of Ce6 fluorescence was observed in A431 cells Figure 3A,D, and these signals were not highly colocalized with the green signal (LysoTracker Green) in the merged images Figure 3C,F. Strong Ce6 fluorescence was detected in A431 cells treated with C225-Ce6/PM Figure 3G, and there was significant colocalization of Ce6 and LysoTracker Green, indicating the accumulation of Ce6 in endosomes/lysosomes and the internalization of C225-Ce6/PM via endocytosis Figure 3I. For HT-29 cells, weak Ce6 fluorescence was observed because of the low expression of EGFR by these cells, and thus it is difficult to determine the extent of Ce6 and LysoTracker Green colocalization Figure 4. These results are consistent with the previous findings presented in Figure 2 and Figure S3.

### 2.3. In Vitro PDT Efficacy of Ce6/PM or C225-Ce6/PM

**Figure 5:**
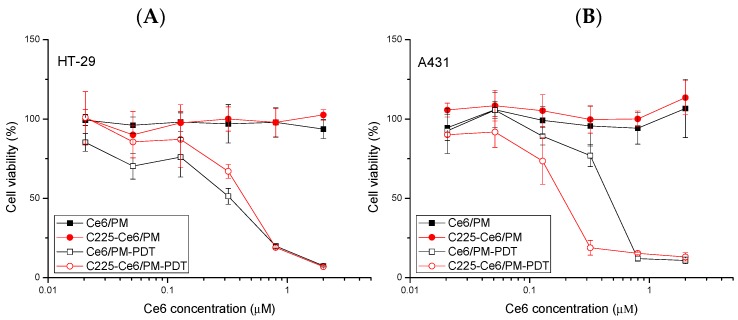
Cytotoxicity and phototoxicity of Ce6/PM and C225-Ce6/PM with or without light irradiation. The light dose was 10 J/cm^2^. (**A**) Low-EGFR-expressing HT-29 cells; (**B**) High-EGFR-expressing A431 cells.

To evaluate the cytotoxicity and phototoxicity of Ce6/PM and C225-Ce6/PM against cancer, A431 or HT-29 cells were treated with micellar photosensitizing agents with or without 10 J/cm^2^ light irradiation. No significant cell toxicities due to Ce6/PM or C225-Ce6/PM were observed in either cell type in the absence of irradiation. Based on the results in Figure 5, it is hypothesized that the amount of C225 on the surface of Ce6/PM does not influence cell viability but can target residues for EGFR-selective drug delivery. For PDT treatments, the IC_50_ value of Ce6/PM-PDT (0.41 μM) in low-EGFR-expressing HT-29 cells was similar to that of C225-Ce6/PM-PDT (0.44 μM). In high-EGFR-expressing A431 cells, C225-Ce6/PM-mediated PDT significantly reduced the cell viability compared with Ce6/PM-mediated PDT, and the IC_50_ value decreased from 0.42 to 0.173 μM, indicating the enhanced killing of EGFR-overexpressing cells. This improvement in the PDT efficacy using a C225-conjugated delivery system was likely due to the increased cellular uptake of Ce6 in A431 cells.

### 2.4. Photodynamic Therapy of Micellar Photosensitizer In Vivo

**Figure 6:**
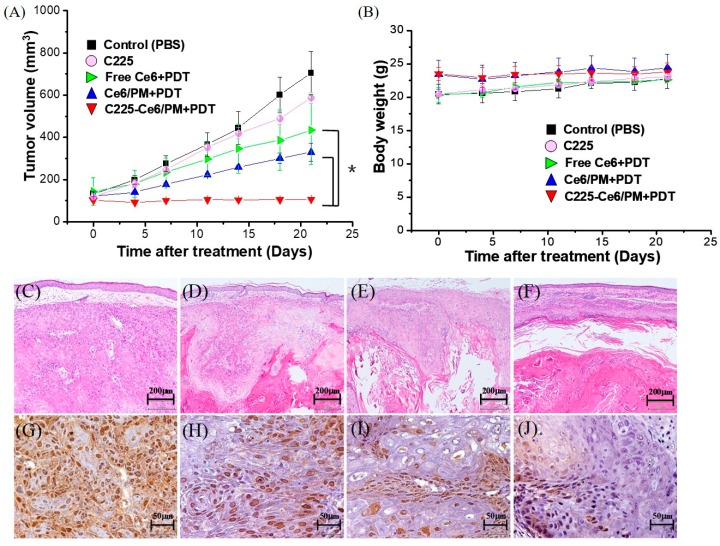
(**A**) Regrowth curves and (**B**) relative weight curves of A431 tumors in nude mice submitted to photodynamic therapy (PDT) treatment. The data are presented as the means ± standard deviation. * Significantly different compared with the control or non-tumor targeting PDT-treated groups (*p* < 0.05). Hematoxylin and eosin (H&E) and proliferating cell nuclear antigen (PCNA) staining of A431 xenograft tumors treated with phosphate-buffered saline (PBS) or micellar Ce6 with irradiation. (**C**,**G**) Control. (**D**,**H**) Ce6-PDT; (**E**,**I**) Ce6-PDT/PM; (**F**,**J**) C225-Ce6/PM-PDT. (**C**–**F**): H&E staining. (**G**–**J**): PCNA staining.

To demonstrate the therapeutic efficacy in vivo, mice were equally divided into five groups: phosphate-buffered saline (PBS) control, C225, Ce6 + PDT, Ce6/PM + PDT, and C225-Ce6/PM + PDT. PBS, free Ce6, Ce6/PM, C225-Ce6/PM (5 mg Ce6 equivalents/kg), and C225 (0.44 mg/mL) were intravenously injected into mice with EGFR-overexpressing A431 tumors, followed by light treatment at 24 h post-injection with a dose of 100 J/cm^2^. Figure 6A shows the relative weight curves of nude mice with or without PDT treatment. The weight differences among the five groups were similar, and no apparent weight loss was observed during the experimental period.

The antitumor efficacies of non-targeted and actively targeted micellar-PDT were evaluated by measuring the tumor growth rates. Figure 6B shows the regrowth curves of EGFR-overexpressing A431 tumors in all groups. For the control group of animals treated with PBS, the tumor volumes continuously increased from day 0 to 21 (PBS-control: from 132.1 ± 11.6 to 704.9 ± 101.4 mm^3^). A similar result was observed in the C225 group, indicating almost no antitumor effect of C225 at this concentration. For the Ce6-PDT and Ce6/PM-PDT groups, the tumor volumes remained stable from days 0 to 3 and then slowly increased from day 3 to day 21. After adjusting for time effects, no significant difference in tumor volume was found between the Ce6-PDT and Ce6/PM-PDT groups. Interestingly, the tumor volumes in the C225-Ce6/PM-PDT group were significantly smaller than those in the Ce6-PDT and Ce6/PM-PDT groups (*p* < 0.05). The percentages of tumor growth inhibition (TGI%) in the Ce6- and Ce6/PM-mediated PDT groups were 38.4 and 53.3, respectively. The smallest tumor size was observed in the C225-Ce6/PM-PDT group (TGI% = 84.8). An equivalent concentration of free C225 did not result in significant tumor suppression compared with the control group, and the PDT efficacy of Ce6 could be significantly improved by using the actively targeted micellar delivery system. C225 and PDT seem to function synergistically to improve cancer treatment.

To evaluate the effects of C225-Ce6/PM-mediated PDT on the EGFR-overexpressing tumor region, tumor specimens from mice sacrificed on day 21 were fixed, embedded, sectioned, and stained. As shown in Figure 6D–J, examination of hematoxylin and eosin (H&E)-stained tissue sections revealed differences in tissue morphology between the treatment groups: the size of the well-differentiated region was clearly decreased in the PDT-treated mice, and only a few viable tumor cells were observed in the C225-Ce6/PM-PDT group (Figure 6F). Cell proliferation in the tumor region was evaluated based on the expression of proliferating cell nuclear antigen (PCNA) as a proliferation marker for immunohistochemical analysis. As shown in Figure 6G–J, mice treated with Ce6-PDT (Figure 6H) or Ce6/PM-PDT (Figure 6I) significantly reduced cell proliferation compared with the level observed in the control group (Figure 6G). Notably, tumors from mice treated with C225-Ce6/PM exhibited a significantly lower rate of cell proliferation Figure 6J. Therefore, actively targeted micelle-based Ce6-PDT can efficiently reduce cell proliferation in the A431 xenograft model.

## 3. Discussion

Conventional PDT has been successfully used in clinics for the treatment both malignant and non-malignant diseases; however, skin photosensitivity is still a common side effect that results in an inconvenient daily life for the duration of therapy. For the traditional hydrophobic photosensitizer formulations using Cremophor, ethanol, or propylene glycol, the undesired partitioning of the photosensitizer into lipoproteins in the blood may result in nonspecific accumulation in undesired tissues, thereby increasing the photosensitivity of these tissues and leading to dose-limiting toxicity. Thus, current PDT needs to be improved if more selective targeting of the photosensitizer is possible. For examples, polymeric micelles provide an excellent platform that can keep hydrophobic photosensitizers within the hydrophobic core without significant drug leakage/partition into lipoproteins, and can passively improve tumor accumulation based on EPR effects [21]. Our previous study demonstrated that poly(2-ethyl-2-oxazoline)-*b*-poly(d,l-lactide) (PEOz-*b*-PLA)-based polymeric micelles significantly reduced the skin phototoxicity of Foscan, and the PDT efficacy was improved by using folate-conjugated polymeric micelles [34,35]. Compared with liposomes of the same size, polymeric micelles formed by amphiphilic copolymers have higher loading capacities for hydrophobic drugs and were sufficiently stable to be used for biomedical applications.

EGF receptor (EGFR) can be detected as overexpressed in some tumors, such as prostate cancer, bladder cancer, breast cancer, ovarian cancer, non-small cell lung cancer, and head and neck cancer, and has also been found to play an important role in the progression of several human malignancies [36]. In previous studies, it was evident that tumors with high EGFR expression seem to be more sensitive to chemotherapeutic agents or radiotherapy. For example, pretreated EGF in overexpression EFGR ovarian cancer cells resulted in increased sensitivity of these cells to cisplatin [37]. Targeting of EGFR with monoclonal antibodies has become feasible with chimeric and humanized antibodies such as cetuximab (C225). C225 acts as a signaling inhibitor via binding to the accessible extracellular domain of EGFR for blocking EGFR activity and inhibiting the unregulated progression of cell cycle [38], and has been approved by the FDA (Food and Drug Administration) for the treatment of a variety of EGFR-positive cancers [39]. Hong and co-workers demonstrated that C225 binds EGFR in tumor tissue and forms EGFR/C225 complexes [40]. Lin et al. used high EGFR expression cell line A549 and low EGFR expression cell line H1299 to explore the therapeutic effects of C225-AuNPs (gold nanoparticles), and their results showed that C225-AuNPs efficiently promoted cytotoxicity and antitumor effect in EGFR-positive cells compared to the C225 alone group [41]. C225-based immunotherapy combined with PDT has been demonstrated to be a synergistic strategy for cancer treatments. Hasan et al. reported that intraperitoneal treatment of liposomal benzoporphyrin derivative monoacid ring A (BPD)-mediated PDT combined with C225 (total dose: 2 mg per mouse) could improve the effectiveness of each approach in a model of human ovarian cancer because of a mechanistically non-overlapping combination modality [42]. Zhang et al. reported on C225-conjugated, gemcitabine-containing magnetic albumin nanospheres as a theranositc nanocarrier to conduct simultaneous targeting, magnetic resonance imaging, double-target thermochemotherapy [43]. Thus, C225 is a potential agent for clinical use in the treatment of EGFR overexpression tumors. In our study, C225-conjugated polymeric micelles for Ce6 delivery were prepared that revealed selectivity of EFGR and light administration, thereby enhancing the antitumor PDT efficacy of Ce6 in EGFR overexpressed cells in vitro (Figure 5) and in vivo (Figure 6). Similar in vitro results were observed using silicon phthalocyanine Pc 4-mediated PDT delivered by EGFR-targeting GE11 peptide-conjugated micelles [44]. Thus, targetable PDT using a nanoscale delivery system can provide a potential platform for efficient and selective cell killing.

Cellular internalization of free Ce6 with three carboxylic chains is considered with emphasis on pH effects [45]. Merlin et al. demonstrated that the intracellular distribution of Ce6 was suggested to be endoplasmic reticulum, Golgi apparatus, mitochondria, and lysosomes based on fluorescence microscopic observation in MCF-7 cells [46]. After micelle encapsulation with C225 conjugation, cellular internalization of micellar Ce6 is suggested to be receptor-mediated endocytosis [29], consistent with our finding as shown in Figure 3. Recently, it has been demonstrated that Ce6 can be successfully formulated using polyvinylpyrrolidone or Pluronics through Van-der-Waals and hydrogen interactions [47,48]. However, self-quenching of singlet oxygen generation and fluorescence of hydrophobic photosensitizers in certain prepared micelles or polymer delivery systems may result in decreased PDT efficacy. The degree of self-quenching is dependent on the extent of the interdigitated hydrophobic interactions between the hydrophobic polymer/photosensitizers or photosensitizers/photosensitizers. The disassociation of these aggregated photosensitizers from the polymeric carriers through the degradation of polymer backbone or linkage broken between drug-polymer revealed the successful PDT ability, and thus this “off-on” strategy can reduce the side effects of PDT [49,50,51]. However, the application of this PDT strategy in clinical settings is difficult, and needs to be investigated in more detail in vivo before clinical trial. In our previous report, we demonstrated micellar photosensitizer delivery systems with unquenched fluorescence property that exhibited less skin phototoxicity in vivo [34,35]. In our study, Ce6 was mainly encapsulated into polymeric micelles through hydrophobic-hydrophobic interactions, and the PDT process of Ce6 in mPEG-*b*-PLA micelles can be directly initiated with light irradiation. It is known that one of the major mechanisms of PDT’s effect in cancer therapy is the photodamage of tumor blood vessels [52], and we found that Ce6/PM-mediated PDT with a 50 J/cm^2^ light dose resulted in efficient vascular shutdown in mouse ears 60 min after intravenous injection (Figure S4). In contrast to the “off-on” PDT strategy, our micelle platform with targeting ligand conjugation provides a suitable environment for hydrophobic photosensitizers encapsulation without self-quenching and does not require the intracellular release of Ce6 from the polymer vehicle, which has the great potential for clinical targetable PDT. Further optimization of the treatment parameters of active targetable photosensitizer/PM-mediated PDT is needed for advanced clinical applications. The combination with other strategies such as light delivery systems, new generation photosensitizers or new approaches for photosensitizer activation are also necessary to achieve satisfactory PDT efficacy in the future.

## 4. Materials and Methods

### 4.1. Materials

The anti-EGFR monoclonal antibody (mAb) C225 was purchased from Merck KGaA (Darmstadt, Germany). The photosensitizer chlorin e6 (Ce6) was obtained from Frontier Scientific, Inc. (Logan, UT, USA). Hydroxyl poly(ethylene glycol)-maleimide (Mal-PEG-OH, Mal-PEG, MW 3400 Da) was purchased from JenKem Technology Co., Ltd. (Allen, TX, USA), and its chemical structure was confirmed by NMR. Monomethoxy poly(ethylene glycol) (CH_3_O-PEG-OH, MW 2000), l-lactide, pyrene, IgG, ZnEt_2_, 3-(4,5-dimethylthiazol-2-yl)-2,5-diphenyltetrazolium bromide (MTT), toluene, methanol, *n*-hexane, tetrahydrofuran (THF), dimethylsulfoxide (DMSO), and dichloromethane (DCM) were purchased from Sigma-Aldrich (St. Louis, MO, USA). Traut’s reagent (2-iminothiolane) was obtained from Pierce (Rockfield, IL, USA).

### 4.2. Preparation of mPEG-b-PLA/Mal-PEG-b-PLA Mixed Micelles with or without Ce6

The synthetic procedure and detailed characterization of diblock copolymer are shown in Figures S5 and S6 and Table S1, and polymeric micelles were prepared by the solvent evaporation method as described in our previous report [53]. Briefly, 10 mg of amphiphilic copolymer (weight ratio of mPEG-*b*-PLA/Mal-PEG-*b*-PLA = 1/9) with or without 1 mg of Ce6 was first dissolved in 3 mL of THF in a sample vial, and the solution was added dropwise into 10 mL of water using a syringe pump. The resulting mixture was stirred for 24 h at 450 rpm, allowing the slow evaporation of the THF and micelle formation. The free activated maleimide groups were blocked using 0.5 μL of 2-mercaptoethanol. The residual THF was completely evaporated at room temperature using a rotary evaporator (N-1000SW, EYELA, Bunkyo-ku, Tokyo, Japan). The resultant solution was filtered through a 0.45 μm filter to remove unencapsulated Ce6 aggregates.

The encapsulation efficiency and the amount of Ce6 in the micelles were determined using a UV-Visible spectrophotometer (U-3000, Hitachi, Japan), and the size of the prepared micelles was measured using a Zetasizer Nano ZS (Malvern Instruments, Worcestershire, UK). The morphology of the micelles was observed by transmission electron microscopy (TEM, JEM-2010, JEOL Ltd., Akishima, Tokyo, Japan).

### 4.3. Preparation of C225- or IgG-Conjugated mPEG-b-PLA/Mal-PEG-b-PLA Mixed Micelles

To conjugate ligands to the micelle surface, C225 and IgG were thiolated for 1 h at room temperature by reacting them with a 20-fold excess of Traut’s reagent and 2 mM EDTA. Ellman’s reagent was used to determine the average number of sulfhydryl groups per thiolated C225 [54]. To synthesize C225-conjugated micelles, a 1:100 molar ratio of thiolated C225 to maleimide moieties in Ce6-loaded polymeric mixed micelles (Ce6/PM) was reacted in aqueous solution for 24 h at 4 °C. Free activated maleimide groups were blocked using 0.5 μL of 2-mercaptoethanol. Unreacted regents and free C225 in the reaction mixture were removed using a 1 × 27 cm Sepharose CL-4B column eluted with 20 mM phosphate at pH 6.5, and C225-conjugated Ce6-loaded micelles (C225-Ce6/PM) were obtained. The concentration of C225 was determined using the Micro BCA Protein Assay Kit (Pierce, Rockfield, IL, USA). IgG-Ce6/PM, a control sample, was prepared using the procedure as for C225-Ce6/PM described above. The hydrodynamic size and the size distribution of the prepared micelles were measured using DLS, and the morphology of the micelles was observed by TEM. 

### 4.4. Cell Culture

A431 cells as the EGFR overexpression cell line [55,56] and HT-29 as the low-EGFR expression cell line [57,58] were used to evaluate the selectivity of the C225-conjugated micelles. Both cells were purchased from the American Type Culture Collection. The cells were maintained in a humidified 5% CO_2_ incubator at 37 °C in DMEM (Gibco BRL, Gaithersburg, MD, USA) supplemented with 10% heat-activated fetal bovine serum (Gibco BRL), 1% sodium pyruvate, and 1% antibiotics (antibiotic-antimycotic, Gibco BRL).

### 4.5. Cellular Uptake of Photosensitizing Agents

To evaluate the cellular uptake of micelles in EGFR overexpressing A431 cells or low-EGFR expression HT-29 cells, 1 × 10^4^ cells/well were seeded into 96-well culture plates for 24 h and then treated with Ce6/PM, C225-Ce6/PM, or IgG-Ce6/PM for another 15, 60, or 240 min at 37 °C. A Ce6 concentration of 5 μM was used to quantify the internalized Ce6 delivered by unconjugated or C225-conjugated micelles. After incubation, the cells were washed twice with 0.1 mL PBS and then lysed in 50 μL of 20% SDS for 24 h to give a homogenous solution. The cell lysates were collected, and the fluorescence intensity was measured using a plate reader (SpectraMax M2e, Molecular Devices LLC, Sunnyvale, CA, USA) with excitation/emission wavelengths of 407/654 nm, respectively. The cellular uptake of Ce6 was normalized to the amount of protein determined using the BCA Protein Assay Kit. Data were obtained from at least three independent experiments. To evaluate micelle selectivity in cells overexpressing EGFR, cells were cultured in medium containing free C225 (0.5 mg/mL) for 30 min prior to the addition of Ce6/PM or C225-Ce6/PM with Ce6 concentrations equivalent to 5 μM to occupy EGFR on the cell surface. These cells were then incubated for another 1 h at 37 °C, and the amount of Ce6 was determined using a SpectraMax M2e reader as described above.

### 4.6. Intracellular Distribution of Ce6/PM or C225-Ce6/PM

To evaluate the intracellular distributions of Ce6/PM and C225-Ce6/PM with or without C225 competition, A431 or HT-29 cells were first seeded onto glass coverslips in 35 mm dishes at a density of 1 × 10^5^ cells per dish. After 24 h of incubation, the cells were washed twice with PBS and incubated with Ce6/PM or C225-Ce6/PM with Ce6 concentrations equivalent to 5 μM for 15 min. For the competition experiment, cells were treated with fresh medium containing C225 (500 μg/mL) for 30 min before incubation with Ce6/PM or C225-Ce6/PM. After incubation, the cells were washed three times with PBS, fixed with 1 mL of formaldehyde, stained using 1 μg/mL Hoechst 33342 (10 mg/mL) for 30 min, and then imaged by CLSM with excitation/emission wavelengths of 405/415–485 nm or 633/650–750 nm, respectively.

To evaluate the endosomal/lysosomal localization of Ce6/PM and C225-Ce6/PM in A431 or HT-29 cells, cells were treated with LysoTracker Green, which was used according to the manufacturer’s protocol, to identify lysosomes. Cultures of these cells were also incubated with Ce6/PM or C225-Ce6/PM (Ce6: 5 µM) for 6 h at 37 °C. The co-localization of LysoTracker Green and Ce6 was evaluated by CLSM.

### 4.7. In Vitro Cell Toxicity and Phototoxicity of Ce6/PM and C225-Ce6/PM

Cells were seeded into 96-well plates at a density of 8 × 10^3^ cells per well and cultured for 24 h. To determine the cytotoxicity of Ce6/PM and C225-Ce6/PM, cells were incubated in medium containing 2.5-fold serial dilutions of Ce6/PM or C225-Ce6/PM from 5 to 0.02048 μM for 4 h at 37 °C. The cells were then washed with PBS and cultured for another 24 h. Then, the cells were washed and subjected to cell viability assays. To determine the PDT effects of the photosensitizer concentration in vitro, cells were incubated with different concentrations of Ce6/PM or C225-Ce6/PM for 4 h at 37 °C, washed, immediately exposed to light (10 J/cm^2^), and then evaluated by cell viability assays after 24 h of incubation. After the addition of photosensitizing agents, all procedures were performed under low light. The light source—consisting of an array of light-emitting diodes (LEDs; covering a spectral region of 650–670 nm with peak intensity at approximately 660 nm)—for activating Ce6 was obtained from ITRI (Hsin Chu, Taiwan), and the fluence rate was 19.5 mW/cm^2^.

The MTT assay to assess cell viability was performed in triplicate using cells seeded onto 96-well plates [59]. After treatment, the culture medium was replaced with fresh medium containing 3-(4,5-dimethylthiazol-2-yl)-2,5-diphenyltetrazolium bromide (MTT), and then the cells were incubated at 37 °C for 2.5 h. Subsequently, the contents of the wells were aspirated to the extent possible without disturbing the formazan crystals and cells on the plastic surface. Finally, 0.1 µL of DMSO was added to each well, and then the plates were placed on a plate shaker for 30 min. Colorimetric measurements were performed using a microplate reader at 570 nm.

### 4.8. Antitumor Efficacy of Ce6/PM and C225-Ce6/PM-Mediated PDT

The in vivo experimental protocols were approved by the Institutional Animal Care and Use Committee (IACUC) of National Chung Hsing University, Taiwan, ROC (IACUC Permit NO. 104-020) based on the Guide for the Care and Use of Laboratory Animals. Female BALB/cAnN.Cg-Foxn1nu/CrlNarl nude mice (4–5 weeks old, 20 ± 2 g) were obtained from the National Laboratory Animal Center (Taiwan). All mice were kept in an air-conditioned facility with an artificial light–dark cycle and were provided standard food and filtered water. The mice were acclimated to this environment for at least three days prior to subcutaneous injection in the right hindquarter with 2 × 10^6^ A431 cells (EGFR-overexpressing cells) suspended in serum-free DMEM. The tumor sizes and body weights were measured every 3 or 4 days for the duration of the experiment. The tumor volume was calculated as 1/2(4π/3)(*L*/2)(*W*/2)*H*, where *L* is the length, *W* is the width, and *H* is the height of the tumor. Treatments were initiated when the tumors reached a volume of approximately 100 mm^3^ (day 0). Mice were randomized into five treatment groups (*n* = 3 per group). The animals were injected with 0.1 mL of PBS (control group), C225, free Ce6, Ce6/PM, or C225-Ce6/PM (Ce6, 5 mg/kg) via the lateral tail vein. Animals that received PBS (vehicle) were used as controls. After 24 h, the tumor was illuminated with a diode laser at 662 nm (100 J/cm^2^) and a light spot diameter of 2 cm. Tumor size and any changes in body weight were recorded for each mouse. The percentage of tumor growth inhibition (TGI%) was calculated from the relative tumor volume at day 21. The PDT-related skin photosensitivity was also evaluated as described in our previous report [34,35]. The left hind leg of each animal was treated with PDT in a manner identical to that in the tumor response studies. Three mice at each drug concentration were evaluated using a 100 J/cm^2^ light dose.

### 4.9. Necropsy and Immunohistochemical Analysis

Tumors were excised after the mice were sacrificed. Prior to immunohistochemical and hematoxylin and eosin (H&E) staining, the tumor tissue was fixed in formalin and embedded in paraffin. Paraffin-embedded 3-μm tumor sections were immunohistochemically assessed for proliferating cell nuclear antigen (PCNA) expression. Briefly, sections were subjected to deparaffinization, rehydrated, and then incubated in 3% H_2_O_2_ to inhibit endogenous peroxidase activity. The sections were then incubated with diluted normal blocking serum to block nonspecific protein binding sites and then with a primary antibody against PCNA (PC10, 1:200, Dako, Denmark). After being rinsed with ddH_2_O, the tissue sections were incubated with the appropriate biotinylated secondary antibody for 30 min at room temperature. The avidin–biotin complexes were visualized with 3,3′-diaminobenzidine tetrahydrochloride (DAB). Sections were also counterstained with hematoxylin. Stained sections were monitored at low power (40×) and counted at high power (400×). Cells in at least five different fields at 400× magnification that stained positively for PCNA were counted. Images of the stained sections were acquired using a light microscope (BX 50, OLYMPUS, Shinjuku-ku, Tokyo, Japan) equipped with a digital camera (DP 20, OLYMPUS, Shinjuku-ku, Tokyo, Japan).

### 4.10. Statistical Analysis

All data are expressed as means ± standard deviation. Tumor volumes for each group measured at different time points are summarized as means and standard deviations. Group effects on tumor volume were tested using a linear mixed model with Bonferroni correction and presented as the estimated marginal means (EM means) and the corresponding 95% confidence interval (CI) of tumor volume, with adjustment for time effects. Statistical significance was set at 0.05. Statistical analyses were performed with the SPSS 15.0 software package (SPSS Inc., Chicago, IL, USA).

## 5. Conclusions

In our research, we successfully developed a C225-conjugated micellar photosensitizer that can be selectively internalized into cancer cells via receptor-mediated endocytosis. In EGFR-overexpressed animal model, this nanoformulation-mediated PDT significantly suppressed tumor growth through the inhibition of cell proliferation. Therefore, these findings suggest that the active EGFR targeting of Ce6-loaded micelles provides a possible way to resolve dose-limiting toxicity of conventional photosensitizers and represents a potential delivery system for PDT in a clinical setting.

## Supplementary Materials

The following are available online at http://www.mdpi.com/2079-4991/8/2/121/s1, Figure S1: The elution profile of Ce6 encapsulated with C225 conjugated mPEG-*b*-PLA/Mal-PEG-*b*-PLA mixed micelles. Figure S2: Images of SDS-PAGE before and after comassie brilliant blue staining. Figure S3: Confocal images of high-EGFR-expressing A431 cells and low-EGFR-expressing HT-29 cells incubated with Ce6/PM or C225-Ce6/ with or without free C225 pretreatment. Figure S4: Vascular observation with different PDT doses after Ce6 or micellar Ce6 postinjection. Figure S5: Ring-opening polymerization of l-lactide for preparation of mPEG-*b*-PLA and Mal-PEG-*b*-PLA diblock copolymer. Figure S6: ^1^H NMR spectra of mPEG-*b*-PLA and Mal-PEG-*b*-PLA diblock copolymer in CDCl_3_. Table S1: The characteristics of synthesized mPEG-*b*-PLA and Mal-PEG-*b*-PLA diblock copolymers.
