# Supplementary material for: Enhanced Antitumor Effects of Epidermal Growth Factor Receptor Targetable Cetuximab-Conjugated Polymeric Micelles for Photodynamic Therapy
Source: Nanomaterials (Basel). 2018 Feb 22;8(2):121. doi: 10.3390/nano8020121 (PMC5853752; doi:10.3390/nano8020121)
Supplement: Supplementary file 1 [file nanomaterials-08-00121-s001.pdf]

Supplementary materials

Enhanced Antitumor Effects of Epidermal Growth Factor Receptor Targetable Cetuximab-Conjugated Polymeric Micelles for Photodynamic Therapy

Ming-Hsiang Chang [1], Chin-Ling Pai [2], Ying-Chen Chen [1], Hsiu-Ping Yu [1], Chia-Yen Hsu [1] and Ping-Shan Lai [1,2,*]

[1] Department of Chemistry, National Chung Hsing University, Taichung 402, Taiwan; s19850309@gmail.com (M.-H.C.); ritachen0623@gmail.com (Y.-C.C.); hsiupingyu0126@gmail.com (H.-P.Y.); clhsqoo@gmail.com (C.-Y.H.); pslai@email.nchu.edu.tw (P.-S.L.)

[2] Ph.D. Program in Tissue Engineering and Regenerative Medicine, National Chung Hsing University, Taichung 402, Taiwan; pailittleelf@gmail.com (C.-L.P.); pslai@email.nchu.edu.tw (P.-S.L.)

* Correspondence: pslai@email.nchu.edu.tw; Tel.: +886-4-22840411-428

Purification and characterization of Cetuximab conjugated mPEG-*b*-PLA/ Mal-PEG-*b*-PLA mixed micelles

After the C225 conjugated on the surface of mixed micelles, a Sepharose CL-4B column (1 × 27 cm) was used to remove the unreacted reagents and unconjugated C225 and 20 mM phosphate at pH 6.5 as elution buffer. The concentration of C225 in each fraction was determined using the Micro BCA Protein Assay Kit (Pierce, Rockfield, IL, USA). The elution profile was shown in Fig. S1. To demonstrate that the C225 was conjugated successfully on the Ce6/PM surface, the collected fraction was evaluated using SDS-PAGE electrophoresis (Fig. S2).

The C225-Ce6/PM (land 6) and Ce6/PM (land 5) have two visible fluorescence bands under UV exposure at the upper region and lower region (Fig. S2C, D). The readable fluorescing signals at the lower region of SDS-PAGE should the Ce6 leakage from the mixed micelles during electrophoresis that were also could be confirmed and comparable with free Ce6 (Fig. S2D: lane 7). The weaker fluorescing signals at the upper region of SDS-PAGE, thus, indicted the Ce6 at this position may still be encapsulated in the integrated mixed micelles (Fig. S2C). In addition, there was a band revealed at the upper region of comassie brilliant blue stained SDS-PAGE under C225-Ce6/PM loading position (Fig. S2B: land 6). To further determined the conjugation of C225 and mixed micelles, a 20% SDS treated C225-Ce6/PM was also evaluated using the same method. As Fig. S2B shown, the C225-Ce6/PM with 20% SDS (lane 9) treated sample revealed a band which was exhibited the same position as C225-Ce6/PM (Fig. S2B: land 6). Taken together, the different mobility of free C225 or thiolated C225 (Fig. S2B: lane 3 and lane 4) with C225-Ce6/PM suggested that the conjugation between thiolated C225 and maleimide decorated mixed micelles was successful and a pure C225-CE6/PM could be acquired via the column chromatography of Sepharose CL-4B.

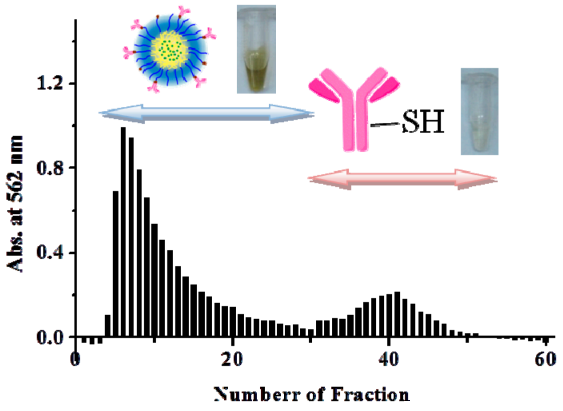

Figure S1. The elution profile of Ce6 encapsulated with Cetuximab conjugated mPEG-*b*-PLA/Mal-PEG-*b*-PLA mixed micelles.

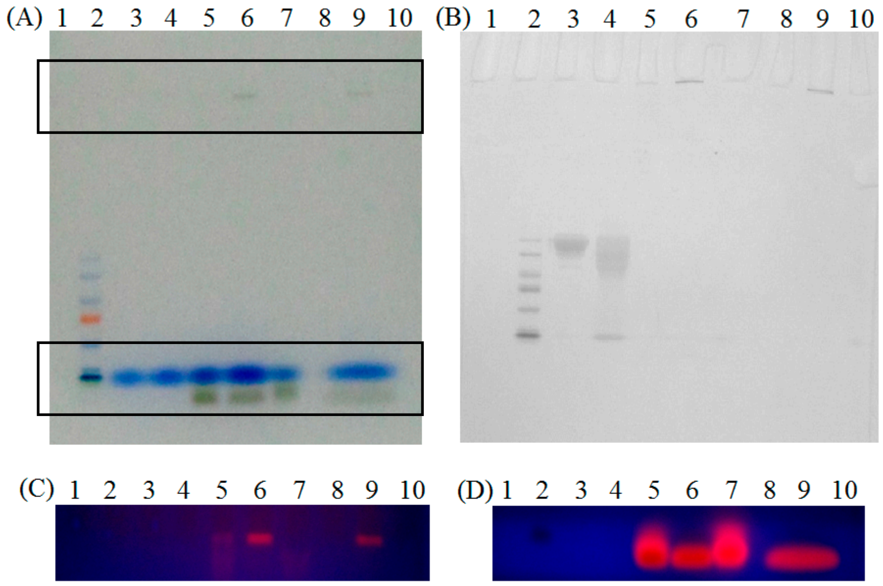

Figure S2. Imaging of SDS-PAGE (**A**) before comassie brilliant blue staining; (**B**) after comassie brilliant blue staining and (**C**. upper region, **D**. lower region) the imaging of SDS-PAGE before comassie brilliant blue staining under UV exposure, where lane 1: none, lane 2: protein marker, lane 3: free C225, lane 4: thiolated C225, lane 5: Ce6/PM, lane 6: C225-Ce6/PM, lane 7: free Ce6, lane 8: none, lane 9: C225-Ce6/PM with 20% SDS, lane10: none.

Confocal microscopic observation of cellular uptake of prepared micelles

To evaluate the intracellular distributions of Ce6/PM and C225-Ce6/PM with or without C225 competition (Fig. S3), A431 or HT-29 cells were first seeded onto glass coverslips in 35 mm dishes at a density of 1 x 10^5 cells per dish. After 24 hours if incubation, the cells were washed twice with PBS and incubated with Ce6/PM or C225-Ce6/PM with Ce6 concentrations equivalent to 5 μM for 15 min. For the competition experiment, cells were treated with fresh medium containing C225 (500 μg/ mL) for 30 min before incubation with Ce6/PM or C225-Ce6/PM. After incubation, the cells were washed three times with PBS, fixed with 1 mL of formaldehyde, stained using 1 μg/ mL Hoechst (10 mg/ mL) for 30 min and then imaged by confocal laser scanning microscopy (Leica-SP5, Leica Microsystems Heidelberg GmbH, Heidelberg, Germany) with excitation/emission wavelengths of 405/415-485 nm or 633/650-750 nm, respectively.

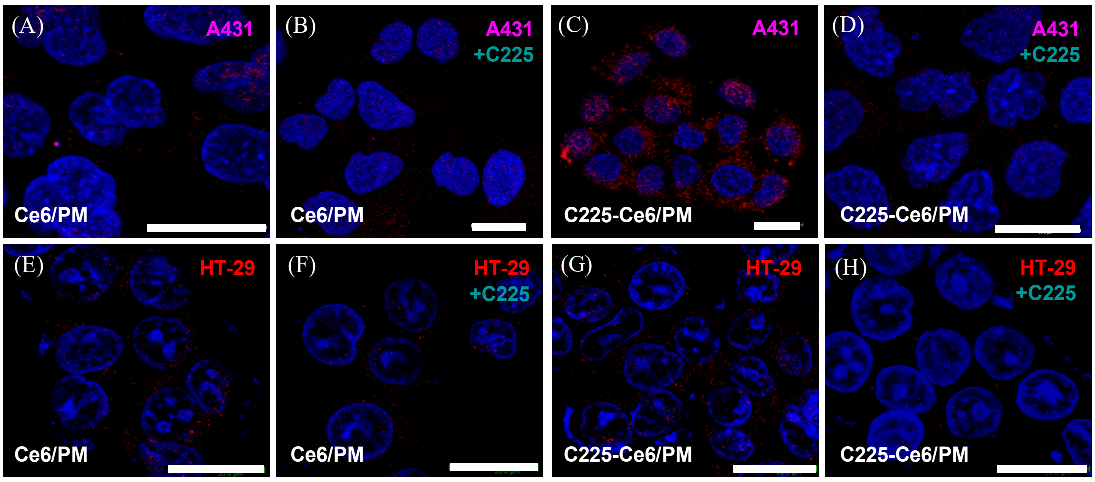

Figure S3. Confocal images of (**A–D**) high-EGFR-expressing A431 cells and (**E–H**) low-EGFR-expressing HT-29 cells incubated with Ce6/PM or C225-Ce6/PM for 60 min with or without free C225 pretreatment (500 μg/ mL for 30 min). Blue color: Hoechst. Red color: Ce6. Scale bar: 20 μm.

Tumor blood vessels photodamage evaluation

It is known that one of the major mechanisms of PDT's effect in cancer therapy is the photodamage of tumor blood vessels [1]. In this experiment, B16F10 cells were subcutaneous injection in the nude mice ears. After 7 days tumor growth, two formulations of free Ce6 or micellar Ce6 were injected via mice tail vein and exposed the mice ears to light irradiation with 25 and 50 J/ cm^2 light dose post 60 min administration (Fig. S4). An efficient vascular shutdown could be observed in mice ears after PDT treatment.

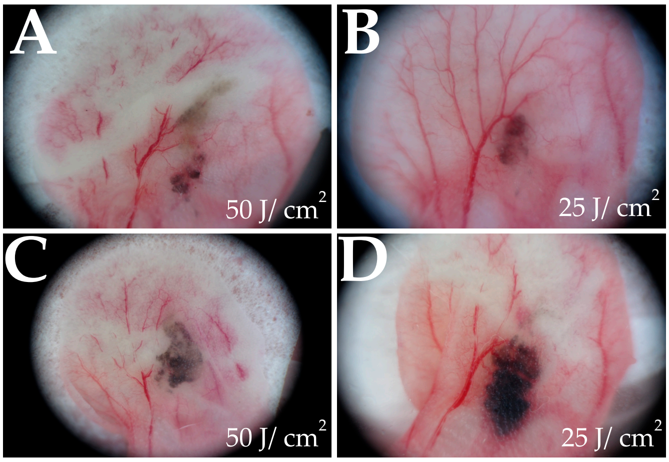

Figure S4. Vascular observation with different PDT doses after 60 min Ce6 or micellar Ce6 postinjection. (**A–B**): free Ce6-mediated PDT; (**C–D**): micellar Ce6-mediated PDT.

Synthesis of mPEG-*b*-PLA and Mal-PEG-*b*-PLA Diblock Copolymers.

To synthesize mPEG-*b*-PLA and maleimide-functionalized poly(ethylene glycol)-block-polylactide (Mal-PEG-*b*-PLA) copolymers, an efficient catalyst, bidentate sulfonamide zinc ethyl complex $[(MPTHQ)ZnEt]_2$, was first prepared by the reaction of 2-(2-methoxyphenyl)-3-tosyl-1,2,3,4-tetrahydroquinazoline (MPTHQ) with $ZnEt_2$ in toluene. For the ring-opening polymerization of L-lactide, a mixture of $[(MPTHQ)ZnEt]_2$ (0.048 g, 0.05 mmol), mPEG (0.400 g 0.200 mmol), and L-lactide in toluene (20.0 mL) was stirred at room temperature for 6

hours. The reaction was quenched by the addition of methanol solution (2.0 mL), and *n*-hexane (50.0 mL) was then added to the above mixture to give a white solid. The white solid was dissolved in DCM and recrystallized using *n*-hexane, and then white crystals were obtained after drying under a vacuum. The Mal-PEG-*b*-PLA copolymer was synthesized using a similar procedure. The polymer structure was characterized by ^{1}H NMR in $CDCl_3$ using a 600 MHz Varian instrument, and the molecular weight distribution was measured by gel permeation chromatography (GPC) with a Waters 410 GPC instrument equipped with two Waters Styragel columns (HT6E, HT3) and a differential refractometer detector. The measurements were taken at 25 °C. THF was used as the eluent at a flow rate of 1.0 mL/ min. The synthetic procedure and detail characterization of diblock copolymer were shown in Fig. S5, S6 and Table S1.

Characterization of mPEG-*b*-PLA and maleimide-PEG-*b*-PLA

The mPEG-*b*-PLA or maleimide-PEG-*b*-PLA (Mal-PEG-*b*-PLA) diblock copolymer was synthesized by ring-opening polymerization [2] of L-lactide in the presence of mPEG or Mal-PEG containing a hydroxyl functional group catalyzed by bidentate sulfonamide zinc ethyl complex (Fig. S5).

The structure of the diblock copolymer was analyzed by ^{1}H NMR in $CDCl_3$ using a 600 MHz Varian instrument. The average number molecular weight (Mn) of the mPEG and Mal-PEG moiety was determined the integrals of the peaks from methoxy proton (δ 3.38 ppm) of mPEG segment and maleimide proton (δ 6.70 ppm) of Mal-PEG segment corresponding to methylene proton (δ 3.65 ppm) of mPEG segment. Similarly, the average number molecular weight (Mn) of the PLA moiety in diblock copolymer was determined the integrals of the peaks from methylene proton (δ 3.65 ppm) of mPGE segment and maleimide proton (δ 6.70 ppm) of Mal-PEG segment corresponding to methine proton (δ 5.17 ppm in mPEG-*b*-PLA and δ 5.16 ppm in Mal-PEG-*b*-PLA) of PLA segment. Molecular distribution (Mw/Mn) were determined by gel permeation chromatography (GPC) which equipped with two Waters Styragel columns using THF as the mobile phase and calibrated with polystyrene standards from molecular weight of 3 to 3150 kDa. The average number molecular weight, molecular weight and molecular distribution (Mw/Mn) of diblock copolymer were determined by ^{1}H NMR and GPC shown in Table S1.

For the preparation of mixed micelles, $mPEG_{2000}$-*b*-PLA_{7400} and Mal-PEG_{3400}-*b*-PLA_{7300} copolymers, which contain more hydrophobic segments and thus can encapsulate more hydrophobic drugs in the inner core of the micelles, were utilized in the following studies. The chemical shift of $mPEG_{2000}$-*b*-PLA_{7400} and Mal-PEG_{3400}-*b*-PLA_{7300} diblock copolymer was shown respectively as below: (600 MHz, $CDCl_3$) δ 5.17 (*q*, *J* = 6.6, Hz, 1H, –C(=O) –CH(–CH_3–)), δ 3.65 (br s, 2H, –OCH_2CH_2–), δ 3.38 (s, 3H, –OCH_3), δ 1.58 (*d*, *J* = 7.2 Hz, 3H, –OCH(–CH_3)C(=O)–) (Fig. S6A) and δ 5.16 (*q*, *J* = 7.2, Hz, 1H, –C(=O)–CH(–CH_3–)), δ 3.65 (br s, 2H, –OCH_2CH_2–), δ 6.70 (s, 2H, –CO–CH=CH–CO–), δ 1.58 (*d*, *J* = 7.2 Hz, 3H, –OCH(–CH_3)C(=O)–) (Fig. S6B). The chemical shifts were in agreement with previously published results on mPEG-*b*-PLA diblock copolymers [3] and on maleimide functionalized PEG-*b*-PLA diblock copolymers [4].

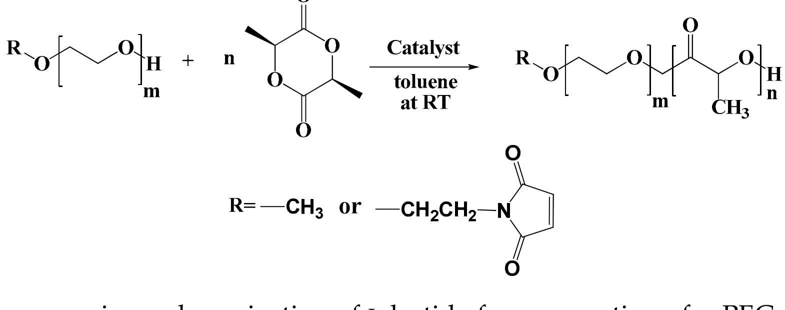

Figure S5. Ring-opening polymerization of L-lactide for preparation of mPEG-*b*-PLA and Mal-PEG-*b*-PLA diblock copolymer.

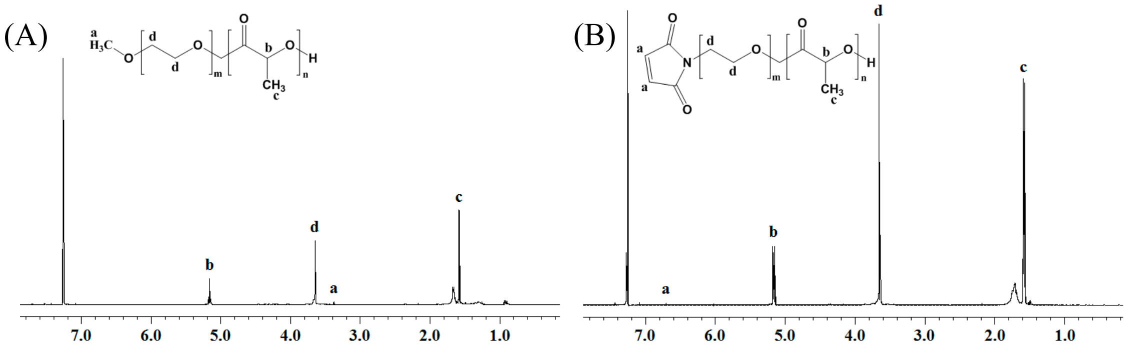

Figure S6. ^{1}H NMR spectra of (A) mPEG$_{2000}$-*b*-PLA$_{7400}$ and (B) Mal-PEG$_{3400}$-*b*-PLA$_{7300}$ diblock copolymer in $CDCl_3$.

Table S1. The characteristics of synthesized diblock copolymers.

Diblock copolymer	Mn (GPC)	Mn (NMR)	Mw/Mn (GPC)
mPEG$_{2000}$-*b*-PLA$_{7400}$	15,800	9,400	1.06
Mal-PEG$_{3400}$-*b*-PLA$_{7300}$	19,300	10,700	1.10

References

1. Chen, B.; Roskams, T.; Xu, Y.; Agostinis, P.; de Witte, P. A., Photodynamic therapy with hypericin induces vascular damage and apoptosis in the RIF-1 mouse tumor model. *Int J. Cancer*. **2002**, *98*, 284-290.
2. Stridsberg, K.; Ryner, M.; Albertsson, A.-C., Controlled Ring-Opening Polymerization: Polymers with designed Macromolecular Architecture. In *Degradable Aliphatic Polyesters*, Springer: Berlin/Heidelberg, Germany, **2002**, *157*, 41–65.
3. Yuan, L.; Xian Rong, Q.; Yoshie, M.; Tsuneji, N., PEG–PLA diblock copolymer micelle-like nanoparticles as all-trans-retinoic acid carrier: *in vivo* and *in vivo* characterizations. *Nanotechnology* **2009**, *20*, 055106.
4. Parikh, T.; Bommana, M. M.; Squillante Iii, E., Efficacy of surface charge in targeting pegylated nanoparticles of sulpiride to the brain. *Eur J Pharm Biopharm* **2010**, *74*, 442-450.
